# Depression among women with obstetric fistula, and pelvic organ prolapse in northwest Ethiopia

**DOI:** 10.1186/1471-244X-13-236

**Published:** 2013-09-26

**Authors:** Berihun Megabiaw Zeleke, Tadesse Awoke Ayele, Mulatu Adefris Woldetsadik, Telake Azale Bisetegn, Akilew Awoke Adane

**Affiliations:** 1 Epidemiology and Biostatistics department, Institute of Public Health, College of Medicine and Health Sciences, University of Gondar, Gondar, Ethiopia; 2 Department of Gynaecology and Obstetrics, School of Medicine, College of Medicine and Health Sciences, University of Gondar, Gondar, Ethiopia; 3 Department of Reproductive Health and Health Education, Institute of Public Health, College of Medicine and Health Sciences, University of Gondar, Gondar, Ethiopia

**Keywords:** Depression, Pelvic organ prolapse, Obstetric fistula, Ethiopia

## Abstract

**Background:**

The prevalence of depression is not well studied among women with pelvic floor disorders. Hence, this study aimed to determine the prevalence of depression and its associated factors among women with pelvic floor disorders.

**Methods:**

A cross-sectional study was conducted among 306 women with one or more of the advanced pelvic floor disorders who attended at the gynaecologic outpatient clinic of Gondar university referral hospital in the six months data collection period. Women who complained of urinary or faecal incontinence or protruding mass per vagina were assessed and staged accordingly. Eligible women i.e. those with advanced pelvic organ prolapse or obstetric fistula were included consecutively. A structured questionnaire was used to obtain socio-demographic data and medical histories for all consenting women. Interviews were done by a female midwife nurse. Depression measures were obtained using the Beck’s Depression Inventory (BDI) tool administered by the midwife nurse after intensive training. Data were entered into a computer using Epi Info version 3. 5.3, and then exported to SPSS version 20 for analysis. Multiple logistic regressions were fitted and Odds ratios with 95% confidence intervals were calculated to identify associated factors.

**Results:**

Of the 306 women interviewed, 269 had advanced pelvic organ prolapse (stages 3 and 4), 37 had obstetric fistula. All four women (100%) with both faecal and urinary incontinence, 97.0% those with urinary incontinence due to obstetric fistula and 67.7% of those with advanced pelvic organ prolapse (stages 3 and 4) had symptoms of depression. Depression was significantly associated with age 50 years or older (P < 0.01), marital status (P < 0.05), history of divorce (p < 0.01), self perception of severe problem (P < 0.05), and having stage 3 pelvic organ prolapse (P < 0.01).

**Conclusion:**

Women with advanced pelvic organ prolapse, and obstetric fistula had high prevalence of depressive symptoms. A holistic management approach, including mental health care is recommended for women having such severe forms of pelvic floor disorders.

## Background

Pelvic floor disorders, mainly urinary and faecal incontinence, and pelvic organ prolapse, having closely related aetiologies, are common among women of developing countries [1]. Pelvic organ prolapse (POP) occurs when the pelvic floor no longer supports the proper positioning of the pelvic organs, i.e. the vagina, bladder, rectum or uterus. Symptoms of POP include a feeling of vaginal bulging and/or pelvic pressure [1]. Women with obstetric fistula are incontinent of urine, faeces, or both. POP, though not uncommon among rural young women [2], is mostly common among older and multiparous women [3], while obstetric fistula among the very young primiparous [4].

Many women with pelvic floor disorders do not seek medical advice [3,5,6] and this makes determining the incidence of gynecological conditions like prolapse and urinary incontinence very difficult. Such gynaecological conditions can impact on sexual well-being of women [7,8] and subsequently psychological problems. Difficulties arise when studying gynaecological morbidities because of the sensitive and hidden nature of complaints regarding of the genital area [9]. In areas where access to health care is often limited, women usually have to live with the consequences of fistula or prolapse for the rest of their lives [3] which can be a challenge, both physically and emotionally, as the symptoms can disrupt the woman’s day-to-day life [7,10].

Failure to control sphincters or having a the uterus outside the vagina, swinging in between thighs, can severely affect a woman’s quality of life by limiting her physical, social, psychological and sexual functions and may cause a great deal of discomfort and distress [3,5,11]. Depressive disorders are common, chronic, and a principal source of disability throughout the world especially among women [12,13]. Prevalence of depression is 3–5 times higher among women with advanced stages of POP compared to normal controls [14,15]. The medical and social consequences of obstetric fistula are distressing and can have a profound effect on psychiatric health [16] since depression has been associated with conditions that often accompany disability [17]. Severe forms of gynaecological conditions such as advanced POP and obstetric fistulae having diverse complications, ranging from personal to social, and majorly contribute to disrupted marital relationships or divorce [7,10].

Reflective of most settings of a developing countries, Ethiopia is prone to a number of maternal health related problems like early marriage (median age at first marriage being 14.2 years [18]), grand-multiparity, obstructed labour and its complications, POP [19] and fistula [20]. In Ethiopia so far there is extremely scarce documented literature on the magnitude of depression among patients seeking care for obstetric fistula and/or POP. Hence, this study assessed the prevalence and identified associated factors of depression among patients with obstetric fistula, and advanced POP.

## Methods

A hospital based cross-sectional study was conducted in a four months period (March 1– June 30, 2012) at the gynaecologic clinic of Gondar University Referral Hospital. Women examined and diagnosed by general practitioners as ‘cases’ of obstetric fistula, or advanced POP were recruited for this study. Prolapse was staged according to the S-POPQ staging system [21]. Fistulae of vesico-vaginal (VVF) and/or recto-vaginal (RVF) which warranted surgery and women who had advanced stages of prolapse i.e. stages 3 and 4. Stage 3 POP represents a stage of prolapse where the leading point descended >1 cm outside the hymenal ring, but does not form a complete vaginal vault eversion while stage 4 is complete vaginal vault eversion or procidentia uteri [21].

Data were collected by a midwife nurse who was regularly working at the clinic. The data collector was trained by the research team on methods of data collection. An interview-based questionnaire was used to collect relevant data. The questionnaire was structured into four parts composed of socio-demography, obstetric and gynaecologic history, symptoms about the prolapse, and symptoms of depression.

Symptoms of depression were screened by Beck’s Depression Inventory (BDI) tool which is composed of 21 questions, each having 0–3 scales. The original BDI tool was translated into Amharic (local language) by two experts independently and then approved by the research team. A woman’s score was summed up out of 63 points and 21 was taken as cut-off point to label ‘symptomatic for depression’.

Data were then entered into a computer using EPI Info Version 3.5.3 and exported to SPSS version 20 for analyzes. Frequency tables and logistic regression tables were used to present results. For risk factor analyses, binary and multivariable logistic regression analyses were performed. All factors with a p-value <0.2 in the bivariate logistic regression were entered into the multivariate model. Odds ratios (OR) with 95% confidence intervals (CI) were calculated to measure associations between the outcome of interest (depression) and explanatory variables. Statistical significance was accepted at the 5% level (p < 0.05).

Ethical clearance was obtained from the University of Gondar ethical review board and an official letter was written to the hospital administration. The study participants were informed about the aims of the study, the confidentiality of the information to be collected. Data were collected after the woman’s verbal consent was obtained.

## Results

### Socio-demographic profile of participants

A total of 306 women with symptomatic pelvic floor disorders (269 POP, 37 VVF of whom 4 had also RVF) were included in this study. Mean age of participants was 50.9 years ±13.3 standard deviations (SDs). The aged of participants ranged from 13–75 years and nearly two-third (63.7%) aged 50 or older (Table 1). Majority (84.0%) were rural residents, orthodox Christians (91.8%) and Amhara by ethnicity (92.6%). More than one in ten (12.3%) of the participant women were currently divorced (Table 1). At an average, women with POP were 23 years older than those with fistula (53.5vs 30.2 years).

**Table 1:** Socio-demographic characteristics of women with advanced pelvic organ prolapse, and fistula at GURH, NORTHWEST Ethiopia (n = 306)

**Characteristic**		**Number**	**Percent**
**Age in years**	22-34	34	11.1
	35-49	80	26.1
	50+	192	62.7
	Mean ± SD	53.5 ± 11.2	
**Residence**	Urban	45	14.7
	Rural	261	85.3
**Religion**	Orthodox Christian	237	91.8
	Muslim	92	8.2
**Ethnicity**	Amhara	284	92.8
	Tigre	22	7.4
**Marital status**	Married	270	88.2
	Divorced	23	7.5
	Widowed	13	4.3
**Educational status**	Unable to read and write	298	97.3
	Read and write or primary	8	2.7
**Occupational status**	House wife	282	92.2
	Employed (gov’t or self)	11	3.6
	Daily Laborer	13	4.2

The mean ages of participants at their first marriage and first delivery were 14.7 years ± 2.2SDs and 17.0 years ± 1.7 SDs, respectively. More than three quarters (79.7%) had at least 5 deliveries. The mean duration of time between occurrence of the problem and reporting to the hospital was 3.0 ± 1.5 years (ranging between 1–10 years). Women with POP sought medical care twice much more later than those with fistula (3.2 vs 1.6 years). Twenty two women (7.2%) had never discussed with anyone about their pelvic floor symptoms before visiting the hospital and about three quarters (77.0%) of those currently married did not discuss the problem with their husband (Table 2).

**Table 2:** Gynaecologic and obstetric history of women with advanced POP, fistula at GURH, northwest Ethiopia, 2012

**Characteristic**		**Number**	**Percent**
Number of deliveries	1	16	5.3
	2-4	46	15.0
	5+	244	79.7
Age at first marriage	<18	288	94.1
	≥18	18	5.9
Age at first delivery	<18	224	73.2
	≥18	82	26.8
History of divorce	Yes	73	23.9
	No	233	76.1
Diagnosis	POP	269	87.9
	Fistula*	37	12.1
Age of occurrence	<20	12	3.9
	20-34	39	12.7
	35-49	109	35.6
	50-64	114	37.3
	65+	32	10.5
	Mean ± SD	48.0 ± 13.1	
Duration of the problem in years	1	38	12.4
	2	98	32.0
	3	68	22.2
	4+	102	32.3

*Four had also recto-vaginal fistula.

### Prevalence and associated factors of depression

A total of 218 women scored 21 or more in the BDI scale, notifying that more than two in three women (71.2%) had depressive symptoms by this screening tool. All the four women with both faecal and urinary incontinence, 97.0% those with urinary incontinence alone, and 67.7% of women with advanced POP (stages 3 and 4) had symptoms of depression. Depression was by far more common among fistula patients than those with POP (97.3% vs 67.7%; p < 0.001).

Symptoms depression were more prevalent among rural than urban women (72.2% Vs 62.1%), divorced than the currently married (91.2% vs 69.1%), and among housewives than employed women (72.3% Vs 45.5%). It was lower among middle aged (34–49 years) women (60%), and those with more advanced POP (65.9% for stage 4 and 76.9% for stage 3 POP).

In the unadjusted analysis, depression as an outcome was significantly associated with age (50 years or older and those younger than 35 years), woman’s level of bother by the problem, and having fistula than POP (Table 3). After adjustment, variables including age 50 years or older (OR 2.06; 95% CI 1.02,4.19), age at first delivery younger than 18 years (OR 2.03; 95% CI 1.07,3.87), marital status (OR 3.41; 95% CI 1.11,10.46), history of divorce (OR 2.25; 95% CI 1.02,4.95), self perception of severe disease (OR 2.30; 95% CI 1.04,5.06), and stage 3 POP (OR 2.75; 95% CI 1.08,7.00) were significantly associated with depression (Table 3).

**Table 3:** Logistic regression analysis of variables with depression among women with advanced pelvic organ prolapse, and obstetric fistula

**Predictor variable**		**Depression**			**Crude OR (95% CI)**	**Adjusted OR (95% CI)**
		**Yes**	**No**	**Total**		
Age	22-34	28	6	34	3.11 (1.16,8.36)*	1.72 (.15,2.45)
	35-49	48	32	80	Reference	Reference
	50+	142	50	192	1.89(1.09,3.29)*	2.06 (1.02,4.19)*
Residence	Urban	28	17	45	Reference	Reference
	Rural	190	71	261	1.63 (.84,3.15)	1.01 (.37,2.73)
Religion	Christian	206	78	284	Reference	Reference
	Muslim	12	10	22	.45(.19,1.09)	.36(.10,1.31)
Currently Marital status	Married	188	82	270	Reference	Reference
	Unmarried	30	6	36	2.18 (.87,5.45)	3.41 (1.11,10.46)*
Age at first delivery	<18	165	59	224	1.53 (.89,2.63)	2.03 (1.07,3.87)*
	18+	53	29	82	Reference	Reference
History of divorce	Yes	55	18	73	1.32(.72,2.39)	2.25(1.02,4.95)*
	No	163	70	233	Reference	Reference
Diagnosis	POP	182	87	269	Reference	Reference
	Fistula	36	1	37	17.23 (2.32,127.58)*	3.45 (0.16,76.90)
Level of bother	Mildly	49	38	87	Reference	Reference
	Moderately	86	35	121	1.91 (1.07,3.40)*	1.38 (.71,2.71)
	Highly	83	15	98	4.29 (2.14,8.59)*	2.30 (1.04,5.06)*
Stage of POP	3	30	9	39	1.72 (.78,3.80)	2.75 (1.08,7.00)*
	4	153	71	232	Reference	Reference

*Significant at P < 0.05.

## Discussion

To our knowledge, this is one of the few studies investigating depressive symptoms in women of developing countries with severe forms of pelvic floor disorders. We studied both women with advanced POP, and women with obstetric fistula and found out a 71.2% prevalence of depression altogether. The prevalence was higher for fistula patients (97.3%).

A 67.7% prevalence of depression is by far higher than the 22.0% prevalence report from the US among women seeking treatment for advanced POP. The US study reported a five times higher prevalence of depression among women with POP compared to those without [14]. Women with advanced forms of POP have decreased body image and overall quality of life [15] compared to their counterparts and this may put them by far for more risks of depression.

A 97.3% prevalence of depression among women with obstetric fistulae (100% for those with both vesico-vaginal and recto-vaginal fistulae) was similar to other reports from Ethiopia (100%) and a report which screened fistula patients from Ethiopia and Bangladish (97%) [22,23]. However, this is higher than a study from Kenya which reported a 72.9% depression symptoms among fistula patients using the Patient Health Questionnaire-9 [16].

Women with fistula were by far more depressed than those with advanced prolapse (97.3% vs 67.7%) though the difference was not significant in the multivariate analysis owing to the lower sample size in one of the groups. Since women living with obstetric fistula experience a deep sense of loss that affected them as women and wives, their social networks are breached and marginalized [10] while women with prolapse are not identified easily. Women with fistula are both socially and personally affected. The fact that genital problems like urinary incontinence and POP are hidden and disgusting for women as reported by previous studies in northwest Ethiopia [8,19] may contribute for the high prevalence of depression in this study.

Similar to other studies [24,25], depression was associated with age extremes of the patient. This is in contrary to a report from breast or gynecologic cancer patients [26] where women younger than 50 were more likely depressed. Currently unmarried women were more depressed than married ones. This may probably be due to the fact that being married is having someone caring and most importantly avoids the social stigma associated with being alone or divorced [10]. Sexual intercourse is often affected, thus putting strain on marital relationships and increasing rates of divorce [7]. This is probably one of the reasons for the high prevalence of depression among women with history of divorce in this study.

This study focused only on patients presenting to the gynaecologic outpatient clinic of a teaching referral hospital, and hence the findings cannot be generalized to all women having similar problems. It is unknown whether women with depressive symptoms may be more bothered and present to the hospital, or the other way round i.e. if women with depressive symptoms are less likely to seek care. As depression has been associated with conditions that often accompany disability and in some cases these disabilities may lead to over or underestimating depression [17,27], the true prevalence of depression might be biased.

Furthermore, employing a cross-sectional study design we cannot make conclusions for some variables in regards to whether depressive symptoms are a risk for or a consequence. This finding likely only hints at the complex interactions between chronic burdens, psychological variables, risk factors and sequelae. Another most important limitation of this study is the use of the BDI which could have overestimated the prevalence of depression due to its focus on somatic symptoms that normally occur in other physical disorders too. The BDI was not validated in Ethiopia although it is widely used in the country as a screening tool for depression.

## Conclusion

Despite limitations, we believe that the study contributes to the existing literature on depression among low income women with severe pelvic floor disorders seeking care and prompts design of interventions for low-income settings. Routine screening (better if using multiple screening tools including a diagnostic depression screen), evaluation, and treatment are strongly recommended since these women are unlikely to receive treatment or supportive counseling at routine outpatient clinics. Additionally, we recommend further studies with qualitative or mixed designs to explore women’s quality of life, and how they experience living with advanced pelvic floor disorders.

## Competing interests

The authors declare that they have no competing interests.

## Authors’ contributions

BMZ wrote the proposal, participated in data collection, analyzed the data and drafted the manuscript. TAA, MAW, TAB and AAA approved the proposal with some revisions, participated in data collection, analysis and manuscript writing. All authors read and approved the final manuscript.

## Pre-publication history

The pre-publication history for this paper can be accessed here:

http://www.biomedcentral.com/1471-244X/13/236/prepub
